# Metadiffusers: Deep-subwavelength sound diffusers

**DOI:** 10.1038/s41598-017-05710-5

**Published:** 2017-07-14

**Authors:** Noé Jiménez, Trevor J. Cox, Vicent Romero-García, Jean-Philippe Groby

**Affiliations:** 1 Laboratoire d’Acoustique de l’Université du Maine - CNRS UMR 6613, Le Mans, 72000 France; 2 Acoustics Research Centre, University of Salford, Salford, M5 4WT United Kingdom

## Abstract

We present deep-subwavelength diffusing surfaces based on acoustic metamaterials, namely *metadiffusers*. These sound diffusers are rigidly backed slotted panels, with each slit being loaded by an array of Helmholtz resonators. Strong dispersion is produced in the slits and slow sound conditions are induced. Thus, the effective thickness of the panel is lengthened introducing its quarter wavelength resonance in the deep-subwavelength regime. By tuning the geometry of the metamaterial, the reflection coefficient of the panel can be tailored to obtain either a custom reflection phase, moderate or even perfect absorption. Using these concepts, we present ultra-thin diffusers where the geometry of the metadiffuser has been tuned to obtain surfaces with spatially dependent reflection coefficients having uniform magnitude Fourier transforms. Various designs are presented where, quadratic residue, primitive root and ternary sequence diffusers are mimicked by metadiffusers whose thickness are 1/46 to 1/20 times the design wavelength, i.e., between about a twentieth and a tenth of the thickness of traditional designs. Finally, a broadband metadiffuser panel of 3 cm thick was designed using optimization methods for frequencies ranging from 250 Hz to 2 kHz.

## Introduction

There are many applications in physics and electrical engineering for objects and surfaces that disperse waves. To take a few examples, such scatterers can be applied to sonar and radar camouflage, electromagnetic reverberation chambers and reducing unwanted ultrasound reflections from surgical equipment. To study how metamaterials might create scattering, this study has focussed on sound diffusers applied in room acoustics. This allows the work to build on a large body of knowledge concerning how such surfaces are measured, predicted and designed. Common wall treatments are made of flat panels, leading to specular sound reflections. In critical environments such as auditoria, professional broadcast and recording control rooms, recording studios or conference rooms, such reflections can decrease sound quality due to echoes or cause sound coloration^1^. Even when these specular reflections are damped by absorption, the sound field inside a room may be non-diffuse, affecting the quality of the listening. In these situations, diffusers can often help by evenly spreading the acoustic energy in both space and time. Specialist diffusers are panels whose scattering function is uniform, so the reflected waves are dispersed in many different directions.

The far-field polar pressure distribution can characterize the performance of a diffuser. For a finite panel of side 2*b*, the far-field polar pressure distribution, *p*_*s*_(*θ*), of a locally-reacting reflecting surface with a spatially dependent reflection coefficient, *R*(*x*), can be calculated using the Fraunhofer integral as ref. 2

1 $$\begin{array}{l}{p}_{s}(\theta )={\int }_{-b}^{b}\,R(x){e}^{j{k}_{0}x\sin \theta }dx,\end{array}$$

where *θ* is the polar angle and *k*_0_ is the wavenumber in air. Note the scattered pressure in the far-field is essentially a Fourier transform of the reflected field along the surface. Therefore, structures whose reflection coefficient distributions present a uniform magnitude Fourier transform present good sound diffusion properties^3^.

**Figure 1:**
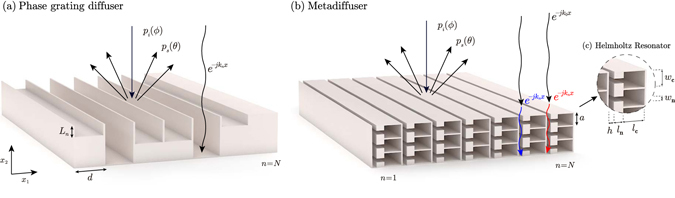
(**a**) Scheme of a QRD Schroeder diffuser composed by *N* = 7 wells or quarter wavelength resonators. (**b**) Metadiffuser composed of *N* = 7 sub-wavelength slits, each of them loaded by *M* = 3 Helmholtz resonators, with slightly different geometry. (**c**) Detail of a slit of the metadiffuser showing the geometrical parameters of the cavity of a HR (*w*_c_ and *l*_c_) and its neck (*w*_n_ and *l*_n_).

The generation of spatially dependent reflective surfaces have been achieved in the past by using phase grating diffusers, also known as Schroeder’s diffusers after its first proposal^3^ using maximum length sequences. The most used configurations are rigid-backed slotted panels where each well acts as a quarter wavelength resonator^4, 5^, as shown in Fig. 1(a). Due to the different resonance frequency of each well, the phase of the reflection coefficient locally depends on the wavenumber and depth of each well. Thus, one approach is to set the spatially-dependent reflection coefficient according to a number sequence that presents a uniform magnitude Fourier spectrum at the design frequency. In this case, a periodic array of the panel presents grating lobes with the same pressure magnitude in the far field at the design frequency.

The maximum phase shift of the reflection coefficient achieved by a single well in a phase grating diffuser occurs at its quarter wavelength resonance, i.e., $L={c}_{0}/4f$ where *f* is the frequency, *L* is the depth of the well and *c*_0_ is the speed of sound in air. Therefore, a limitation of Schroeder diffusers is that the depth becomes large for low design frequencies. This results in thick and heavy panels, limiting the use of phase grating diffusers for low-frequencies where the wavelength of sound in air is of the order of several meters. In the context of smart building design and sustainable building, leading-edge technologies can be applied to optimize space and design lightweight materials, improving the performance of the acoustic solutions using less resources.

Various approaches have been carried in the past to reduce the total thickness of the panels. As the wells of Schroeder diffusers present different lengths, well folding strategies have been proposed^6–8^ to minimize the unused space. At high frequencies the sound does not bend through the folded wells and so care in design is needed. Using well-folding the total thickness of a diffuser can only be reduced to about half the depth of a standard Schroeder diffuser. Other approaches include the use of single Helmholtz resonators instead of quarter wavelength resonators to construct the phase grating diffuser. This strategy was first reported by placing perforated sheets at the front of a Schroeder diffuser^9^. The added-mass effect reduces the resonance frequency of the well and consequently the thickness can be reduced. In this system losses are inevitably introduced and therefore, some of these devices were proposed as sound absorbers^10^. Using “T” shaped wells, 2 dimensional resonators can be designed and the full structure can be optimized to extend its low frequency response^1^. Flat panels have been also proposed using hybrid surfaces that combine patches of absorption and reflection^11^, but their performance is limited because of weak edge diffraction. Other approaches include the use of active surfaces^12^, but their use is limited due to cost. Recently, sonic crystals (SC) were used to construct acoustic diffusers^13^. A SC is a periodic arrangement of acoustic scatters, typically rigid bars embedded in air. The periodicity leads to a modification of the dispersion relations and propagation through these structures becomes strongly dispersive and anisotropic. The diffusion was achieved at low frequencies of a bi-periodic SC mainly caused by the internal Fabri-Pérot resonances of the structure. The main drawback of this promising approach is the lack of simple and/or analytical methods to design these complex structures. Therefore, optimization of these structures have been proposed^14^, but the lack of fast analytical models make the design tedious and, until now, the inherent thermo-viscous losses have not been accounted for in these designs.

Local resonances have also been exploited to introduce strong dispersion in acoustic metamaterials^15^. In these structures the phase speed can be strongly modified and materials with exotic properties as either negative effective bulk modulus or negative mass density^16, 17^ can be designed. Metamaterials have been widely used to design acoustic absorbers as metaporous materials^18–21^, dead-end porosity materials^22, 23^ or absorbing resonant metamaterials composed by membrane-type resonators^17, 24–26^, quarter-wavelength resonators (QWRs)^23, 27–29^ and Helmholtz resonators (HRs)^26, 30–32^. These last types of metamaterials^23, 27, 28, 31, 32^ make use of strong dispersion giving rise to slow-sound propagation inside the material. Using slow sound results in a decrease of the cavity resonance frequency and, hence, the structure thickness can be drastically reduced to the deep-subwavelength regime^31^. Moreover, these structures can fulfil the critical coupling conditions^26^, having their impedance matched with the exterior medium and resulting in perfect absorption (PA), as recently demonstrated for panels using slow sound and QWRs^28^ or HRs^31^.

In this paper, we present deep-subwavelength diffusers based on acoustic metamaterials to reduce the thickness of Schroeder diffusers. The system works as follows: first, we consider a rigid panel of finite length with a set of *N* slits. Second, we modify the dispersion relations inside each slit by loading one of their walls with a set of HRs, as shown in Fig. 1(b). The sound propagation in each slit becomes strongly dispersive and the sound speed inside it, *c*_*p*_, can be drastically reduced. Each slit behaves effectively as a deep-subwavelength resonator and, therefore, the effective depth of the slits can be strongly reduced as $L={c}_{p}/4f$ holds. By tuning the geometry of the HRs and the thickness of the slits, the dispersion relations inside each slit can be modified. As a result the phase of the reflection coefficient can be tailored, e.g., to those of an Schroeder phase grating diffuser. Furthermore, by tuning the thermo-viscous losses, which are inherent in the HRs and in the narrow slits, the leakage of the structure can be compensated by the intrinsic losses of the system and PA can be obtained. Thus, the magnitude of the reflection coefficient can be also tuned, and the behaviour of the slits ranges from perfect reflectors to perfect absorbers. Perfect absorbing slits allows the construct of ternary sequence diffusers^33^ for low frequencies.

## Results

### Slow sound and dispersion relations in the slits

We consider a 2D flat panel composed of a periodic distribution of unit cells. As shown in Fig. 1(b,c), the unit cell is composed by *N* slits of width *h* separated a distance *d* and distributed in the *x*_1_ direction. Each slit is loaded by *M* HRs separated a distance *a*, each one composed of a squared cross-section neck and a cavity with length and width dimensions *l*_*n*_ and *w*_*n*_, *l*_*c*_ and *w*_*c*_ respectively. The propagation inside each slit was calculated using the transfer matrix method (TMM) and the finite element method (FEM) including the thermoviscous losses by means of its effective parameters (see methods section). The methods and unit cell used in this work are the same as in refs 31 and 32. In those works, the TMM and FEM using the effective parameters were accurately validated experimentally to model the thermoviscous losses of metamaterials using a set of *N* = 13 by *M* = 1 HRs^31^ and *N* = 3 by *M* = 10 HRs^32^.

**Figure 2:**
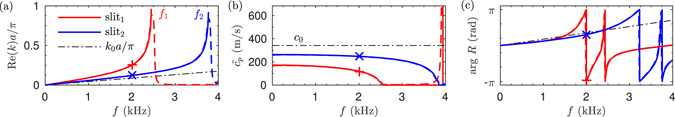
(**a**) Dispersion relation inside the (blue) first and (red) second slits of a metadiffuser for the lossless case (continuous lines) and accounting for the thermo-viscous losses (dashed lines), and wavenumber in air (dashed-dotted). The resonance frequencies of the HR are shown as *f*_1_ and *f*_2_. (**b**) Corresponding phase speed. (**c**) Phase of the reflection coefficient for each individual slit.

Figure 2(a) shows the dispersion relations inside two different slits, *n* = 1 and *n* = 2, obtained by using *M* = 2 HR with the geometrical parameters listed in Table 1. First, above the resonance frequency of the HRs, *f*_*n*_, a band gap is generated. Below the resonance frequency of the HRs a dispersive band is observed and the wavenumber is increased with respect to the wavenumber in air. In this regime, slow sound conditions are produced, as shown in Fig. 2(b), i.e., the phase speed inside the slits is strongly reduced. The phase of the reflection coefficient produced by each slit is shown in Fig. 2(c). We can see that for some frequencies the phase of the reflection coefficient of both slits (blue and red lines) is strongly modified when compared to the reflection phase of a slit without HRs (dashed line). At 2 kHz, the 1st slit (red curve) reacts inverting the phase of the incoming wave, while for the 2nd slit (blue curve) this occurs at 3.2 kHz. In this way, by tuning the geometry a specific phase profile can be tailored, while the total thickness of the panel can be greatly reduced when compared with a quarter wavelength resonator of length *L*. By using these features, we show in this article that the phase profile of Schroeder and ternary sequence diffusers can be mimicked by a sub-wavelength metadiffuser in a given frequency band. Therefore by tuning the geometry of a metadiffuser we can maximize sound diffusion in a broad frequency band for room acoustics applications using a deep sub-wavelength panel.

**Table 1:** Geometrical parameters of the QR-metadiffuser.

*n*	*s*_*n*_	*h* (mm)	*l*_n_ (mm)	*l*_c_ (mm)	*w*_n_ (mm)	*w*_c_ (mm)
1	1.0	14.7	13.0	16.4	6.2	9.0
2	4.0	30.9	9.1	4.3	3.5	9.0
3	4.0	30.9	9.1	4.3	3.5	9.0
4	1.0	15.7	13.3	17.0	6.3	9.0
5	0.0	20.3	18.0	20.7	3.2	9.0

### Quadratic residue metadiffusers

**Figure 3:**
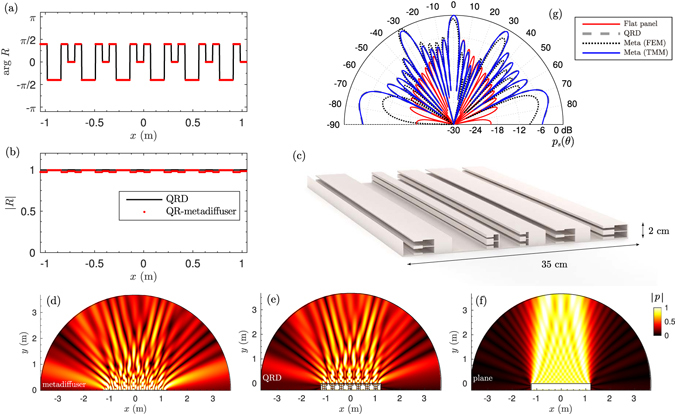
(**a**) Phase and (**b**) magnitude of the spatially-dependent reflection coefficient of a QRD (black line) and the QR-metadiffuser (red doted). (**c**) Scaled scheme of the QR-metadiffuser with *N* = 5 and *M* = 2. (**d**) Near field pressure distribution at 2 kHz of QR-metadiffuser with thickness *L* = 2 cm (**e**) phase grating QRD of thickness *L* = 27.4 cm and (**f**) flat plane reflector. (**g**) Far-field polar distribution of the QR-metadiffuser obtained by TMM (continuous blue) and FEM (dotted black), the reference QRD (dashed-grey), and a plane reflector with same width of the diffusers (continuous red).

The first numerical sequence mimicked is the one used in quadratic residue diffusers (QRD). The sequence is given by *s*_*n*_ = *n* ^2^ mod *N*, where mod is the least non-negative remainder of the prime number *N*. If the phase grating diffuser is based on quarter wavelength resonators (wells), the depth of the wells is given by ${L}_{n}={s}_{n}{\lambda }_{0}/2N$, where *λ*_0_ is the design wavelength. Here, we use optimization methods, e.g., sequential quadratic programming^34^, to tune the geometry of the metamaterial so the spatially-dependent reflection coefficient matched between the QR-metadiffuser and the QRD only at 2000 Hz. A QRD with *N* = 5 QRD, a total thickness of *L* = 27.4 cm and side *Nd* = 35 cm was designed for a frequency of 500 Hz. Due to the small lateral size of the panel, the response was evaluated at 2000 Hz considering 6 repetitions of the unit cell in order to clearly generate the characteristic *N* diffraction grating lobes of the QRD in the far-field. Figure 3(a,b) shows the phase and magnitude of the reflection coefficient along the surface the ideal QRD and a QR-metadiffuser of *L* = 2 cm thickness and *M* = 2 HRs of same lateral dimensions. The geometrical parameters for the metadiffuser are listed in Table 1 and a scheme of the panel is shown in Fig. 3(c). Perfect agreement is found between the reflection coefficients of the QR-metadiffuser and the target phase grating QRD. Figure 3(g) shows the far-field calculation at 2000 Hz using Eq. (1) for both structures. Excellent agreement is obtained between the polar responses using the TMM. To validate the design a full-wave numerical solution using the finite element method (FEM) and accounting for the thermo-viscous losses is also provided. The FEM numerical solution agrees with the theoretical prediction, although small discrepancies can be observed. They are caused first because the radiation corrections used in the TMM are only approximate, and, second, because the evanescent coupling between near slits in the TMM is not considered while it is implicitly included in the FEM simulations. The near field pressure distributions are shown in Fig. 3(d–f) for the QR-metadiffuser, the QRD and a reference flat surface of the same width, respectively. Excellent agreement is observed between both diffusers, where it is clear how the field is scattered in other directions rather than specular. The presented QR-metadiffuser is 17.1 times thinner than the QRD (34 times smaller than the QRD design wavelength (500 Hz) and 8.5 times smaller than the evaluation wavelength (2000 Hz).

### Primitive root metadiffusers

**Figure 4:**
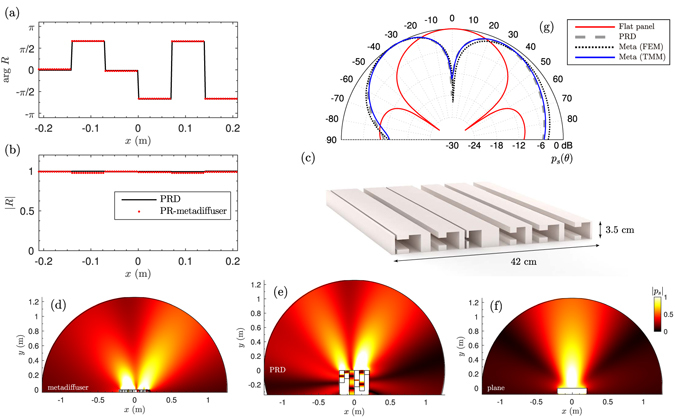
(**a**) Phase and (**b**) magnitude of the spatially-dependent reflection coefficient of a PRD (black line) and the PR-metadiffuser (red doted). (**c**) Scaled scheme of the metadiffuser using *N* = 6 and *M* = 1. (**d**) Near field pressure distribution at 1 kHz of a PR-metadiffuser with prime number *P* = 7 and thickness *L* = 3.5 cm (**e**) phase grating QRD of thickness *L* = 17.1 cm and (**f**) reference flat plane reflector. (**g**) Far-field polar distribution of the PR-metadiffuser obtained by TMM (continuous blue) and FEM (dotted black), the reference PRD (dashed-grey), and a plane reflector with same width of the diffusers (continuous red).

The second numerical sequence presented here is the primitive root sequence, given by *s*_*n*_ = *r* ^*n*^ mod *P*, where *P* is a prime number and *r* is the primitive root of *P*. The primitive root sequence have *N* = *P* − 1 different values. A primitive root diffuser (PRD) is constructed using a set of *N* wells with depths ${L}_{n}={s}_{n}{\lambda }_{0}/2N$. The scattered field by these diffusers presents a notch at specular directions at multiples of the design frequency^4^. Figure 4(a,b) show both the phase and magnitude of the spatially-dependent reflection coefficient of a *P* = 7 phase grating PRD of thickness *L* = 17.1 cm with *d* = 7 cm, and for a PR-metadiffuser of *L* = 3.5 cm and *M* = 1 HR with the same lateral dimensions. Excellent agreement is found between both responses. The corresponding geometrical parameters of the PR-metadiffuser are listed in Table 2, while a scaled scheme is drawn in Fig. 4(c). Figure 4(d–f) show the near field corresponding to the PR-metadiffuser, the PRD, and the reference plane surface. The characteristic notch is observed at normal reflection angle, i.e., *θ* = 0. Note, because the structures are not symmetric, neither is the field. The far-field is presented in Fig. 4(g), where good agreement is found between the theory and the full-wave numerical solutions. Both panels produce the same scattering, but the thickness of the PR-metadiffuser is around 10 times thinner than the phase grating PRD (20 times smaller than the design wavelength).

**Table 2:** Geometrical parameters of the PR-metadiffuser.

*n*	*s*_*n*_	*h* (mm)	*l*_n_ (mm)	*l*_c_ (mm)	*w*_n_ (mm)	*w*_c_ (mm)
1	1.5	0.5	26.1	23.4	19.4	34.0
2	1.0	14.4	16.7	26.0	14.7	34.0
3	3.0	1.1	5.7	26.2	7.3	34.0
4	2.0	22.0	14.3	19.1	13.9	34.0
5	2.5	14.6	18.6	24.7	14.7	34.0
6	0.5	22.4	14.9	19.9	18.3	34.0

### Absorption in QR- and PR-metadiffusers

**Figure 5:**
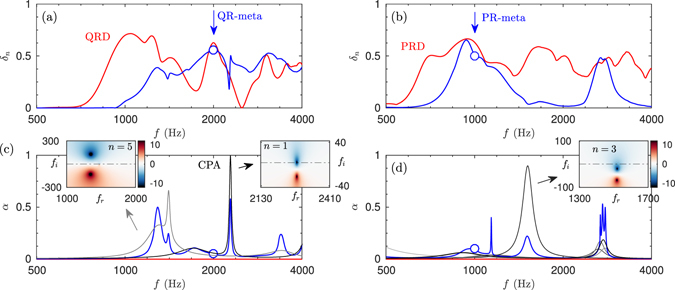
(**a**) Diffusion coefficient of the QR-metadiffuser (blue) optimized at 2000 Hz (marker) and a reference QRD (red). (**b**) Diffusion coefficient of the PR-metadiffuser (blue) optimized at 2000 Hz (marker) and a reference PRD (red). (**c**) Corresponding absorption for the QRD case, where the grey lines shows the absorption of individual slits *n* = 1 and *n* = 5. For these slits, the insets show the complex frequency representation^26^ of the reflection coefficient (log|*R*(*f*_*r*_, *f*_*i*_)|^2^), where *f*_*r*_ = Re(*f*) and *f*_*i*_ = Im(*f*). (**e**) Corresponding absorption for the PRD where the complex frequency representation of the reflection coefficient is shown for the individual slit *n* = 1 in the inset.

These metamaterials show high flexibility to tailor their reflection response to a specific spatial function. However, the presented QR- and PR-metadiffusers are tuned to fit the desired phase response only at a single frequency. In order to quantify the frequency dependent performance of a diffusing panel, the normalized diffusion coefficient, *δ*_*n*_, can be evaluated from the far-field polar responses (see methods section). This parameter measures the uniformity of the scattering pattern, i.e., a high value indicates that there is no privileged reflection direction, zero indicates that the energy is reflected only in one direction. The frequency dependent diffusion coefficient is shown in Fig. 5(a,b) for the QR- and PR- metadiffusers respectively. Although the phase of the reflection coefficient of the metadiffusers does not follow the QRD and PRD design for all frequencies, the surface impedance still varies spatially and creates dispersion. Note, the magnitude of the reflection coefficient is strongly spatially dependent. Compared with the corresponding Schroeder’s diffusers, the magnitude of the diffusion coefficient is of the same order at the design frequency. In the case of the QR-metadiffuser a broadband diffusion is observed when compared with the PR-metadiffuser. This broadband diffusion is mainly achieved by the multiple collective modes of the HRs^32^ produced by a higher *M* value.

In all the previous results, thermo-viscous losses were accounted for in the ducts that comprise the metamaterial. The acoustic absorption due to these thermo-viscous losses is shown in Fig. 5(c,d) for the QR- and PR-metadiffusers respectively. It can be observed that for some frequencies, peaks of absorption are generated (blue curves in Fig. 5(c,d)). Moreover, if the absorption of each individual slit is calculated, very sharp peaks of absorption appear at selected frequencies, as those marked by the arrows (grey curves in Fig. 5(c,d)). For the case of the QR-metadiffuser, at *f* = 2270 Hz the reflection coefficient vanishes at the *n* = 1 slit because it is impedance matched with the exterior air and the critical coupling condition fulfils. The complex frequency plane representation of the eigenvalues of the scattering matrix, i.e., the reflection coefficient, is shown in the insets for the slits *n* = 5 and *n* = 1. The different resonances can be identified with zero-pole pairs in the complex frequency plane. In the case of *n* = 5, the zeros of the eigenvalue of the scattering matrix are close to the real frequency axis and, therefore, it produces a peak of absorption. In the case of *n* = 1 slit, we can observe that the eigenvalues of the scattering matrix present a zero which is exactly located on the real frequency axis. Therefore, at this particular frequency perfect absorption is achieved. It is worth noting here that these structures were first proposed as perfect acoustic absorbers^31^. A similar behaviour is observed for the case of the PRD diffuser at *f* = 1510 Hz, but in this case, imperfect absorption is achieved as also shown in the inset of Fig. 5(d) where the zero of the eigenvalues of the scattering matrix is not exactly on the real frequency axis. Thus, in this case, the critical coupling conditions are not fulfilled.

### Hybrid metadiffusers

The induced absorption can be used to obtain diffusion. Perfect absorption is mandatory to design diffusers based on index^35^, ternary or quadriphase^33^ sequences. The family of ternary sequence diffusers^33^ are based on numerical sequences composed by 3 possible states, [−1, 0, 1], organized in such a way that the magnitude of its Fourier spectrum is uniform. Schroeder’s diffusers based on these sequences use phase gratings (quarter wavelength resonators) to obtain the inverted phase reflection, [−1] state, flat surfaces for the in-phase reflection, $[1]$ state, and high absorptive materials for the zeros of the sequence, $[0]$ state. This can be achieved by filling a well with porous absorbent such as mineral wool. Even when these devices are constructed with long wells, the main limitation is that the reflection does not vanish at low and medium frequencies, due to the poor impedance matching of the rigidly-backed porous material with the air: the porous material enters in the viscous frequency regime and inside it a diffusion-dominated wave equation is satisfied.

**Figure 6:**
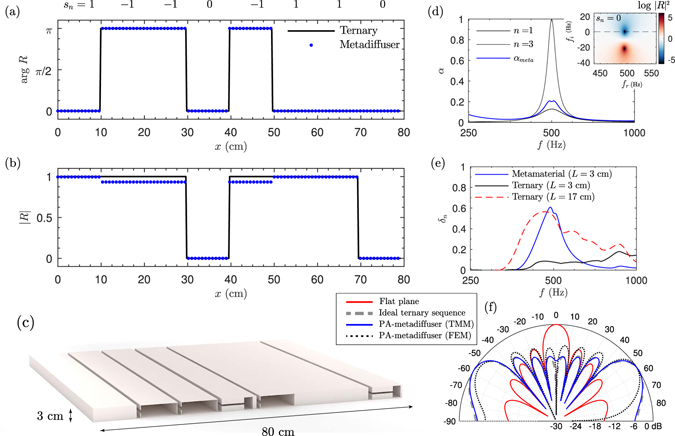
(**a**) Phase and (**b**) magnitude of the spatially dependent reflection coefficient for an ideal ternary sequence diffuser (black) and a PA-metadiffuser (blue dots). The ternary sequence used, *s*_*n*_, is shown on top. (**c**) Scaled scheme of the geometry of the hybrid PA-metadiffuser. (**d**) Frequency dependent absorption for the total structure (blue curve) and individual slits (black curves). The inset shows the complex frequency plane representation of the reflection coefficient for the perfect absorber slits, *s*_*n*_ = 0. (**e**) Frequency dependent diffusion for the PA-metadiffuser of *L* = 3 cm (blue), a ternary sequence diffuser using phase gratings of *L* = 17 cm (dashed red) and ternary sequence diffuser using phase gratings of *L* = 3 cm (black). (**f**) Far field polar response at 500 Hz of a ternary sequence with *N* = 8 wells (dashed-grey), the PA-metadiffuser obtained by TMM (continuous black) and FEM (dotted black), and a plane reflector of the same width as the diffusers (red line).

The use of metadiffusers offers the possibility of accurately creating both the inverted phase and the zeros of ternary sequences: the geometry of the system can be tuned to obtain sub-wavelength wells with inverted phase and perfect absorbers (PA)^31^. In addition, the optimization process is simplified because only 2 different sub-wavelength wells are required with independence of the length of the sequence. A PA-metadiffuser was designed using *N* = 8 and *M* = 1. The phase inverted and perfect absorbers have been obtained by tuning the geometry of the metamaterial using optimization methods with the constraint of *L* < 3 cm, i.e., a panel thickness 23 times smaller than the wavelength at *f* = 500 Hz. The retrieved parameters are listed in Table 3 and a scaled scheme of the metadiffuser is shown in Fig. 6(c). Figure 6(a,b) show the sequence, *s*_*n*_, used to design a ternary sequence diffusers and the corresponding phase and magnitude of the reflection coefficient. Small discrepancies can be observed between the ideal and the calculated spatially dependent reflection coefficient, mainly caused by the inherent absorption of the phase-inverter slits. When the metadiffuser becomes deep-subwavelength, the small ducts that compose the metamaterial lead to unavoidable thermoviscous losses, mainly localized at the neck of the HRs. In contrast, the perfect absorbing slits are accurately obtained. Figure 6(d) shows the frequency dependent absorption of each slit and the total absorption produced by the metadiffuser. The eigenvalues of the scattering matrix in the complex frequency plane are shown in the inset for the PA slit. It can be observed that the eigenvalues of the scattering matrix present a zero that is located exactly on the real frequency axis. Under these conditions the material is critically coupled to the exterior medium and at this particular frequency sound is perfectly absorbed. Figure 6(f) shows the far-field pressure distribution of an ideal ternary sequence diffuser, a PA-metadiffuser using TMM and its corresponding FEM simulation accounting for the thermo-viscous losses. The characteristic notch in the polar response at the specular direction is obtained because the magnitude of the first component of the Fourier spectrum of the used ternary sequence is zero. Only small discrepancies are observed caused by the non-perfect phase inverting slits due to the thermo-viscous losses that appear in this deep-subwavelength thickness structure. The frequency dependent diffusion coefficient is shown in Fig. 6(e). Due to the fact that the metamaterial only present PA at the design frequency, the diffusion coefficient presents a high value only in a narrow frequency band. Note the corresponding correlation scattering coefficient^1^ is almost one, reaching a value of σ_*c*_ = 0.996, indicating that specular reflection almost vanishes. Using PA other sequences with flat Fourier spectrum can also be mimicked, including binary maximum length sequences^3^ or complex Legendre sequences based on the index function^35^.

**Table 3:** Geometrical parameters of the PA-metadifuser.

*s*_*n*_	*h* (mm)	*l*_n_ (mm)	*l*_c_ (mm)	*w*_n_ (mm)	*w*_c_ (mm)
1	0	—	—	—	—
−1	8.5	1.8	88.7	8.4	29.0
0	10.0	69.4	10.2	2.4	29.0

### Broadband optimal metadiffusers

**Figure 7:**
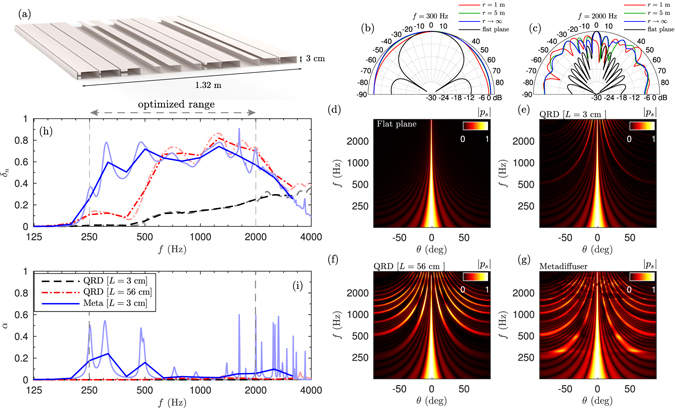
Far field polar response as a function of the frequency for (**a**) reference flat plane, (**b**) *N* = 11 QRD panel with a total thickness of 3 cm, (**c**) QRD panel with total thickness of 56 cm and (**d**) optimized metadiffuser thickness of 3 cm. (**e**) Normalized diffusion coefficient or the 3 cm QRD (dashed black), 32 cm QRD (dashed-dotted red) and optimized metadiffuser using TMM (blue) integrated in third of octaves. The third octave integration is shown in thick lines according to ISO 17497-2:2012^43^. (**f**) Corresponding absorption.

To design a metadiffuser useful for room acoustics, its diffusion must be broad in frequency. Thus, we extended the bandwidth of the optimization procedure, where the cost function to minimize was $\varepsilon =1-{\int }_{{f}_{{\rm{low}}}}^{{f}_{{\rm{high}}}}\,{\delta }_{n}df$. In particular, we look for deep-subwavelength thickness metadiffusers that present maximum normalized diffusion coefficient in the frequency range from *f*_low_ = 250 Hz to *f*_high_ = 2000 Hz. Here, we used a set of *N* = 11 slits separated by *d* = 12 cm, and constrained the thickness of the panel to *L* = 3 cm. The obtained geometrical parameters are listed in Table 4. Here we used square cross-section HRs. Figure 7(a) shows the scheme of the metadiffuser with the retrieved geometry. First, the polar responses at two frequencies, 300 and 2000 Hz are shown in Fig. 7(b,c). The maximization of the diffusion coefficient implies that the polar responses are uniform. In addition, we show the angular dependence of the near field at shorter distances, e.g., at 1 and 5 m. Due to the lateral dimension of the structure is 1.32 m, Eq. (1) is not accurate at distances much shorter than the Rayleigh distance. However, although the near field does not exactly follow the polar distribution given by Eq. (1), the structure scatters the waves uniformly in broad range of angles when compared with a flat plane of same dimensions. See Supplementary material for details about the near field produced by this structure. Figure 7(d–g) show the frequency dependent polar responses in the far field for a reference flat plane with the same width than the metadiffuser, a thick QRD with a design frequency of 250 Hz (*L*_QRD_ = 56 cm), a thin QRD with the same thickness of the metadiffuser *L*_QRD,thin_ = 3 cm, and the optimized metadiffuser, respectively. Here, we calculated the polar responses using 6 repetitions of the panel to clearly observe the diffraction grating lobes. First, the scattering of the thin QRD, Fig. 7(e), is almost the same as a flat plane, Fig. 7(d). It only starts to scatter waves at different angles above 2 kHz. Second, the deep wells that compose the thick QRD, Fig. 7(f), resonate near their quarter-wavelength resonances at lower frequencies and, therefore, the reflection coefficient follows the QR sequence and the panel scatters sound waves into oblique angles. Finally, the optimized metadiffuser, Fig. 7(g), also shows strong grating lobes, but, in addition, at medium and high frequencies energy is spread in other directions at low frequencies, e.g., between 250 and 500 Hz.

**Table 4:** Geometrical parameters used for the broadband metadiffuser using *N* different slits.

*n*	1	2	3	4	5	6	7	8	9	10	11
*h* (mm)	5.7	4.9	7.7	82.9	48.4	74.9	20.0	6.6	76.2	29.5	7.6
*l*_**n**_ (mm)	16.3	7.3	37.1	0.0	35.3	22.1	14.7	0.1	0.0	0.1	4.8
*l*_**c**_ (mm)	97.1	106.8	74.2	36.0	35.3	22.1	84.3	112.2	42.7	89.4	106.5
*w*_**n**_ (mm)	6.7	6.5	10.0	29.0	29.0	29.0	14.0	9.5	29.0	27.6	6.2
*w*_**c**_ (mm)	29.0	29.0	29.0	29.0	29.0	29.0	29.0	29.0	29.0	29.0	29.0

The normalized diffusion coefficient shown in Fig. 7(h) quantifies this behaviour. It is observed that over the optimized range the diffusion coefficient of the metadiffuser takes values with a mean value of about *δ*_*n*_ = 0.65, with peaks of *δ*_*n*_ = 0.9. When compared to the thick QRD, its frequency band is extended to one octave below. The corresponding absorption is shown in Fig. 7(f). Here, the wide slits that form the QRD produce almost no losses, while the thermo-viscous losses produced in the narrow ducts that comprise the ultra-flat metamaterial lead to some peaks of absorption at the resonance of the cavities. These losses can be reduced if the thickness of the panel is increased, but here we presented a structure whose thickness is 46 times smaller than the wavelength. It is worth noting here that the size of some of neck of the resonators is almost the same as their cavities, as can be observed in Fig. 7(a). In these cases the resonator acts as a coiled QWR and the losses in these wide ducts are decreased. The resonance frequency of these QWR is higher than the corresponding HRs, contributing to the high frequency diffusion, while, in contrast, the HRs introduce spatial changes on the reflection coefficient at low frequencies. Moreover, the position of the low frequency absorption peaks can be engineered to solve other typical problems in room acoustics, as placing them at the resonant modes of small control rooms to produce a flatter spectral response, or reduce sound coloration in the reverberation. This can be achieved using multi-objective optimization techniques.

## Discussion

*Metadiffusers*, a novel design of locally reacting surfaces with tailored acoustic scattering was presented. These new structures are based on metamaterials comprising a slotted panel, with the slits loaded by a set of Helmholtz resonators. The propagation inside the metamaterial presents strong dispersion and the sound speed can be significantly reduced so that each slit effectively behaves as a deep-subwavelength resonator. Thus, by tuning the material geometry, the dispersion of acoustic waves in the slits is modified and the spatially-dependent reflection coefficient can be tailored to specific functions with uniform magnitude Fourier transform. In these conditions, the grating lobes produced by a periodic arrangement of the panel all have the same energy. The acoustic energy can be scattered in other directions than specular. Different designs were presented based on number-theoretical sequences as quadratic residue and primary root sequences (QR and PR-metadiffusers). Moreover, using the concept of critical coupling, sub-wavelength perfect absorbers were introduced to accurately model ternary sequence metadiffusers (PA-metadiffusers). Finally, it was shown that the structures can be optimized to work in a broad frequency range covering 3 octaves. In particular, we presented a diffuser of 3 cm thickness working from 250 to 2000 Hz, demonstrating the potential of the metadiffusers to be used in critical listening environments due to their deep-subwavelength nature: the thickness of the panels was 1/46 to 1/20 times the design wavelength, i.e., between about a twentieth and a tenth of the thickness of traditional designs. In the context of smart building design and sustainability, metadiffusers can be used to save space and to produce lightweight materials, improving the performance of the acoustic solutions using less resources. Moreover, the proposed designs have the potential to meet the aesthetic requirements that are mandatory for modern auditoria design.

While the focus of the study has been sound diffusers for rooms, dispersed, broadband reflections are of interest beyond architectural acoustics. Example of structures creating diffuse reflections are found in nature, for example *Cyphochilus* and Lepidiota stigma beetles have chitin networks that achieve an exceptionally bright white colour from all observation angles^36^. A second example would be the use of acoustic camouflage by insects to avoid predation by bats. The latest research suggests that insects look for rough surfaces, ones that create dispersion, to reduce the chances of being detected via echolocation^37^. We would anticipate applications for deliberately designed dispersive surfaces: in underwater acoustics; in airborne acoustics and for other wave types (e.g. light, seismic waves). As in nature, applications might involve signalling, reducing interference from unwanted reflections and acoustic camouflage.

## Methods

### Transfer matrix method

The system described before has been theoretically modelled by using the transfer matrix method. Under the assumption of plane waves travelling inside the metamaterial, either the transfer matrix or the scattering matrix can be obtained, providing directly the reflection of the metamaterial, as well as its effective parameters.

The transfer matrix is used to relate the sound pressures and normal acoustic particle velocities at the beginning and at the end of each slit. The transfer matrix of the *n*-th slit, **T** ^*n*^, of length *L*, extending from *y* = 0 to *y* = *L* is written as

2 $${[\begin{array}{c}{P}^{n}\\ {V}^{n}\end{array}]}_{y=0}={{\bf{T}}}^{n}\,{[\begin{array}{c}{P}^{n}\\ {V}^{n}\end{array}]}_{y=L}=[\begin{array}{cc}{T}_{11}^{n} & {T}_{12}^{n}\\ {T}_{21}^{n} & {T}_{22}^{n}\end{array}]\,{[\begin{array}{c}{P}^{n}\\ {V}^{n}\end{array}]}_{y=L}.$$

For an identical set of *M* resonators, the transmission matrix **T** ^*n*^ is written as

$${{\bf{T}}}^{n}=[\begin{array}{cc}{T}_{11}^{n} & {T}_{12}^{n}\\ {T}_{21}^{n} & {T}_{22}^{n}\end{array}]={{\bf{M}}}_{{\rm{\Delta }}{l}_{{\rm{slit}}}}^{n}\,{({{\bf{M}}}_{{\bf{s}}}^{n}{{\bf{M}}}_{{\rm{HR}}}^{n}{{\bf{M}}}_{{\bf{s}}}^{n})}^{M}.$$

Here, the transmission matrix for each lattice step in the *n*-th slit, ${{\bf{M}}}_{s}^{n}$, is written as

3 $${{\bf{M}}}_{{\bf{s}}}^{n}=[\begin{array}{cc}\cos \,({k}_{{\bf{s}}}^{n}\tfrac{a}{2}) & i{Z}_{{\bf{s}}}^{n}\,\sin \,({k}_{{\bf{s}}}^{n}\tfrac{a}{2})\\ \tfrac{i}{{Z}_{{\bf{s}}}^{n}}\,\sin \,({k}_{s}^{n}\tfrac{a}{2}) & \cos \,({k}_{{\bf{s}}}^{n}\tfrac{a}{2})\end{array}],$$

where the slit characteristic impedance is written as ${Z}_{{\bf{s}}}^{n}=\sqrt{{\kappa }_{{\bf{s}}}^{n}{\rho }_{{\bf{s}}}^{n}}/{S}_{{\bf{s}}}^{n}$ and ${S}_{{\bf{s}}}^{n}={h}^{n}\,a$. The resonators are introduced as punctual scatters by a transmission matrix ${{\bf{M}}}_{{\rm{HR}}}^{n}$ as

4 $${{\bf{M}}}_{{\rm{HR}}}^{n}=[\begin{array}{cc}1 & 0\\ \mathrm{1/}{Z}_{{\rm{HR}}}^{n} & 1\end{array}],$$

and the radiation correction of the *n*-th slit to the free space as

5 $${{\bf{M}}}_{{\rm{\Delta }}{l}_{{\rm{slit}}}}^{n}=[\begin{array}{cc}1 & {Z}_{{\rm{\Delta }}{l}_{{\rm{slit}}}}^{n}\\ 0 & 1\end{array}],$$

with the characteristic radiation impedance of the *n*-th slit ${Z}_{{\rm{\Delta }}{l}_{{\rm{slit}}}}^{n}=-i\omega {\rm{\Delta }}{l}_{{\rm{slit}}}^{n}{\rho }_{0}/{\varphi }_{t}^{n}{S}_{0}$, where *S*_0_ = *da*, *ρ*_0_ the air density and ${\rm{\Delta }}{l}_{{\rm{slit}}}^{n}$ the proper end correction that will be described later.

Finally, the reflection coefficient of the rigidly backed slit can be directly calculated from the elements of the matrix **T** ^*n*^ as

6 $${R}^{n}=\frac{{T}_{11}^{n}-{Z}_{0}{T}_{21}^{n}}{{T}_{11}^{n}+{Z}_{0}{T}_{21}^{n}}.$$

with ${Z}_{0}={\rho }_{0}{c}_{0}/{S}_{0}$, and finally the absorption as ${\alpha }^{n}=1-{|{R}^{n}|}^{2}$. The effective parameters of each slit can be obtained from the transfer matrix elements as follows

7 $${k}_{{\rm{eff}}}^{n}=\frac{1}{L}\,{\cos }^{-1}\,(\frac{{T}_{11}^{n}+{T}_{22}^{n}}{2}),\quad {Z}_{{\rm{eff}}}^{n}=\sqrt{\frac{{T}_{12}^{n}}{{T}_{21}^{n}}}.$$

In the case of different HRs, the total transfer matrix of the whole system can be obtained by the product of the transfer matrices of each layer of the material. Thus, the total transfer matrix method of the system is given by

$${{\bf{T}}}^{n}=[\begin{array}{cc}{T}_{11}^{n} & {T}_{12}^{n}\\ {T}_{21}^{n} & {T}_{22}^{n}\end{array}]={{\bf{M}}}_{{\rm{\Delta }}{l}_{{\rm{slit}}}}^{n}\prod _{m=1}^{M}\,({{\bf{M}}}_{{\bf{s}}}^{n}{{\bf{M}}}_{{\rm{HR}}}^{n,m}{{\bf{M}}}_{{\bf{s}}}^{n}).$$

where the matrix ${{\bf{M}}}_{{\rm{HR}}}^{n,m}$ is calculated for each *m* resonator in each *n* slit.

### Visco-thermal losses model

The visco-thermal losses in the system are considered both in the HRs and in the slits by using its effective complex and frequency dependent parameters. Considering only plane waves propagate inside the metamaterial, the effective parameters of the ducts that conform 2D resonators and the slits of width 2*r* are given by ref. 38:

8 $${\rho }_{{\rm{eff}}}={\rho }_{0}{[1-\frac{\tanh (r{G}_{\rho })}{r{G}_{\rho }}]}^{-1},$$

9 $${\kappa }_{{\rm{eff}}}={\kappa }_{0}\,{[1+(\gamma -\mathrm{1)}\frac{\tanh (r{G}_{\kappa })}{r{G}_{\kappa }}]}^{-1},$$

with ${G}_{\rho }=\sqrt{i\omega {\rho }_{0}/\eta }$ and ${G}_{\kappa }=\sqrt{i\omega {\rm{\Pr }}{\rho }_{0}/\eta }$, and where *γ* is the specific heat ratio of air, *P*_0_ is the atmospheric pressure, Pr is the Prandtl number, *η* the dynamic viscosity, *ρ*_0_ the air density and *κ*_0_ = *γP*_0_ the air bulk modulus. The effective parameters of the *n*-th main slit, ${\rho }_{{\bf{s}}}^{n}$ and ${\kappa }_{{\bf{s}}}^{n}$, are obtained by setting *r* = *h* ^*n*^/2 in Eqs (8 and 9). The visco-thermal losses inside the 2-dimensional resonator’s neck and cavity are modelled in the same way by these effective parameters, ${\rho }_{{\bf{n}}}^{n,m}$, ${\kappa }_{{\bf{n}}}^{n,m}$ and ${\rho }_{{\bf{c}}}^{n,m}$, ${\kappa }_{{\bf{c}}}^{n,m}$ respectively, by setting $r={w}_{{\bf{n}}}^{n,m}\mathrm{/2}$ and $r={w}_{{\bf{c}}}^{n,m}/2$ for the *m*-th resonator located at the *n*-th slit.

### Resonator impedance and end corrections

Using the effective parameters for the neck and cavity elements given by Eqs (8 and 9), the impedance of a Helmholtz resonator, including a length correction due to the radiation can be written as ref. 39:

10 $${Z}_{{\rm{HR}}}^{n,m}=-i\tfrac{\cos ({k}_{{\bf{n}}}{l}_{{\bf{n}}})\,\cos ({k}_{{\bf{c}}}{l}_{{\bf{c}}})-{Z}_{{\bf{n}}}{k}_{{\bf{n}}}{\rm{\Delta }}l\,\cos ({k}_{{\bf{n}}}{l}_{{\bf{n}}})\,\sin ({k}_{{\bf{c}}}{l}_{{\bf{c}}})/{Z}_{{\bf{c}}}-{Z}_{{\bf{n}}}\,\sin ({k}_{{\bf{n}}}{l}_{{\bf{n}}})\,\sin ({k}_{{\bf{c}}}{l}_{{\bf{c}}})/{Z}_{{\bf{c}}}}{\sin ({k}_{{\bf{n}}}{l}_{{\bf{n}}})\,\cos ({k}_{{\bf{c}}}{l}_{{\bf{c}}})/{Z}_{{\bf{n}}}-{k}_{{\bf{n}}}{\rm{\Delta }}l\,\sin ({k}_{{\bf{n}}}{l}_{{\bf{n}}})\,\sin ({k}_{{\bf{c}}}{l}_{{\bf{c}}})/{Z}_{{\bf{c}}}+\,\cos ({k}_{{\bf{n}}}{l}_{{\bf{n}}})\,\sin ({k}_{{\bf{c}}}{l}_{{\bf{c}}})/{Z}_{{\bf{c}}}},$$

where (note superscripts were omitted for the sake of simplicity) ${l}_{{\bf{n}}}^{n,m}$ and ${l}_{{\bf{c}}}^{n,m}$ are the neck and cavity lengths, ${k}_{{\bf{n}}}^{n,m}$ and ${k}_{{\bf{c}}}^{n,m}$, are the effective wavenumbers and and ${Z}_{{\bf{n}}}^{n,m}$ and ${Z}_{{\bf{c}}}^{n,m}$ effective characteristic impedance in the neck and cavities respectively, and Δ*l* ^*n*,*m*^ the correction length for the HRs. These correction lengths are deduced from the addition of two correction lengths ${\rm{\Delta }}{l}^{n,m}={\rm{\Delta }}{l}_{1}^{n,m}+{\rm{\Delta }}{l}_{2}^{n,m}$ as

11 $${\rm{\Delta }}{l}_{1}^{n,m}=0.41\,[1-1.35\frac{{w}_{{\bf{n}}}^{n,m}}{{w}_{{\bf{c}}}^{n,m}}+0.31\,{(\frac{{w}_{{\bf{n}}}^{n,m}}{{w}_{{\bf{c}}}^{n,m}})}^{3}]\,{w}_{{\bf{n}}}^{n,m},$$

12 $${\rm{\Delta }}{l}_{2}^{n,m}=0.41\,[1-0.235\tfrac{{w}_{{\bf{n}}}^{n,m}}{{w}_{{\bf{s}}}^{n}}-1.32\,{(\tfrac{{w}_{{\bf{n}}}^{n,m}}{{w}_{{\bf{s}}}^{n}})}^{2}+1.54\,{(\tfrac{{w}_{{\bf{n}}}^{n,m}}{{w}_{{\bf{s}}}^{n}})}^{3}-0.86\,{(\tfrac{{w}_{{\bf{n}}}^{n,m}}{{w}_{{\bf{s}}}^{n}})}^{4}]\,{w}_{{\bf{n}}}^{n,m}.$$

The first length correction, ${\rm{\Delta }}{l}_{1}^{n,m}$, is due to pressure radiation at the discontinuity from the neck duct to the cavity of the Helmholtz resonator^40^, while the second ${\rm{\Delta }}{l}_{2}^{n,m}$ comes from the radiation at the discontinuity from the neck to the principal waveguide^41^. This correction only depends on the radius of the waveguides, so it becomes important when the duct length is comparable to the radius, i.e., for small neck lengths and for frequencies where ${k}_{{\bf{n}}}^{n,m}{w}_{{\bf{n}}}^{n,m}\ll 1$.

Another important end correction comes from the radiation from the slits to the free air. The radiation correction for a periodic distribution of slits can be expressed as ref. 42:

13 $${\rm{\Delta }}{l}_{{\rm{slit}}}^{n}={h}^{n}{{\rm{\sigma }}}^{n}\sum _{n=1}^{\infty }\,\frac{{\sin }^{2}\,(n\pi {{\rm{\sigma }}}^{n})}{{(n\pi {{\rm{\sigma }}}^{n})}^{3}}.$$

with σ^*n*^ = *h* ^*n*^/*d*. Note for 0.1 ≤ σ^*n*^ ≤ 0.7 this expression reduces to ${\rm{\Delta }}{l}_{{\rm{slit}}}^{n}\approx -\sqrt{2}\,\mathrm{ln}\,[\sin \,(\pi {{\rm{\sigma }}}^{n}\mathrm{/2})]/\pi$.

### Diffusion coefficient

The diffusion coefficient^43^, *d*_*ϕ*_, is estimated from a polar response as

14 $${\delta }_{\varphi }=\frac{{({\int }_{-\pi }^{\pi }{I}_{s}(\theta )d\theta )}^{2}-{\int }_{-\pi }^{\pi }{I}_{s}{(\theta )}^{2}d\theta }{{\int }_{-\pi }^{\pi }{I}_{s}{(\theta )}^{2}d\theta },$$

where *I*_*s*_(*θ*) is the polar scattering intensity for a wave with incident angle *ϕ*. This coefficient is normalized to that of a plane reflector, *δ*_flat_, to eliminate the effect of the finite size of the structure as ${\delta }_{n}=({\delta }_{\varphi }-{\delta }_{{\rm{flat}}})/(1-{\delta }_{{\rm{flat}}})$.

### Finite element simulations

In order to validate the results we use a numerical approach based on the Finite Element Method (FEM) using COMSOL Multiphysics 5.2™. The thermo-viscous losses were accounted for using the effective parameters (complex phase speed and complex density) given by Eqs (8 and 9) for each domain. Rigid boundary conditions were considered at the external sides of the panel and viscous losses were neglected here. This is justified because the losses are mainly produced at the narrow slits that conform the metamaterial and the contribution of other sources is minor. Absorbing boundary conditions (a perfectly matched layer) with a thickness of *λ*_0_, were placed at the boundaries of the numerical domain. The unstructured grid was designed ensuring a maximum element size of *λ*_0_/20. As usual, to obtain the scattering of the panel a background pressure field was set as initial condition in the main domain and the scattered field was computed. By measuring the scattered field over a closed contour the far-field can be obtained^1^.

## Electronic supplementary material

Supplementary material
