# Supplementary material for: Metadiffusers: Deep-subwavelength sound diffusers
Source: Sci Rep. 2017 Jul 14;7:5389. doi: 10.1038/s41598-017-05710-5 (PMC5511165; doi:10.1038/s41598-017-05710-5)
Supplement: Supplementary file 1 — Supplementary material [file 41598_2017_5710_MOESM1_ESM.pdf]

Supplementary material to: Metadiffusers: Deep-subwavelength sound diffusers

Noé Jiménez[1,*], **Trevor J. Cox**[2], **Vicent Romero-García**[1], **and Jean-Philippe Groby**[1]

[1]Laboratoire d'Acoustique de l'Université du Maine - CNRS UMR 6613, Le Mans, 72000 France
[2]Acoustics Research Centre, University of Salford, Salford M5 4WT, United Kingdom
[*]noe.jimenez@univ-lemans.fr

ABSTRACT

In this document we present the Supplementary material for the paper entitled "Metadiffusers: Deep-subwavelength sound diffusers".

Contents

1 Near field of the broadband metadiffuser 1
2 Dependence of the metadiffusers with the incidence angle 1
3 Parameter sensitivity 3

1 Near field of the broadband metadiffuser

To extend the diffusing performance to low frequencies, the slits that compose any diffuser must be broadly spaced in the lateral dimension. In addition, to achieve the broadband behaviour of the broadband metadiffuser, with a high number of resonances, the number of slits of the last metadiffuser was selected as $N = 11$, leading to a large panel whose lateral dimension was 1.32 m. The enlargement of the lateral dimensions allows the realization of low-frequency diffusion. On the other hand, the Fourier-Fraunhofer integral (Eq. (1)) is only accurate in the far-field, i.e., distances much longer than $2b$. However, at realistic distances for room acoustics, the receivers of the waves scattered by this large panel should be in the near field. To test the validity of the results, the near field produced by the broadband metadiffuser was also calculated at realistic distances, e.g., at 1, 2 and 5 m from the structure. Figure 1 summarizes the results. Essentially, when the polar responses are evaluated at lower distances, although Eq. (1) is not valid, the field is scattered by the structure with a polar distribution that is roughly similar than the far field polar response. Note that the fact that Eq.(1) is not accurate does not imply that the waves in the field are not scattered in a diffuse way. These results show that the large size of the structure does not limit the application of metadiffusers in practice.

2 Dependence of the metadiffusers with the incidence angle

Like phase grating diffusers, the structures presented here are locally reacting surfaces. Acoustic waves cannot propagate inside the structure from slit to slit and, therefore, the surface impedance of the structure, Z_e, do not depend on the angle of the incident wave. For these structures the angle-dependent reflection coefficient is then given by

$$R(\phi) = \frac{Z_e - Z_0/\cos\phi}{Z_e + Z_0/\cos\phi}. \tag{1}$$

Therefore, no special differences are expected from the traditional phase grating diffuser designs. Figure 2 shows the performance of the diffusers as a function of the incidence angle, ϕ. First, the normalized diffusion coefficient for the QR-metadiffuser is shown in Fig. 2 (a). The diffusing performance of its equivalent Schroeder's QRD follows the angular dependence of the metadiffuser. As expected, the diffusion coefficient inversely depends on the number of repetitions of the panel, as periodicity introduces strong grating lobes in the polar response. Second, the corresponding diffusion coefficient as a function of the incidence angle for the PR-metadiffuser and the reference Schroeder's PRD is shown in Fig. 2 (b). As observed in the QR-metadiffuser, no special differences exist between the metadiffuser and the traditional diffuser designs. Of special interest is the case of the PA-metadiffuser. Perfect absorbers built using metamaterials based on slow sound provides quasi-omnidirectional absorption?. For this reason, the scattering coefficient of ternary sequence PA-metadiffusers show weak

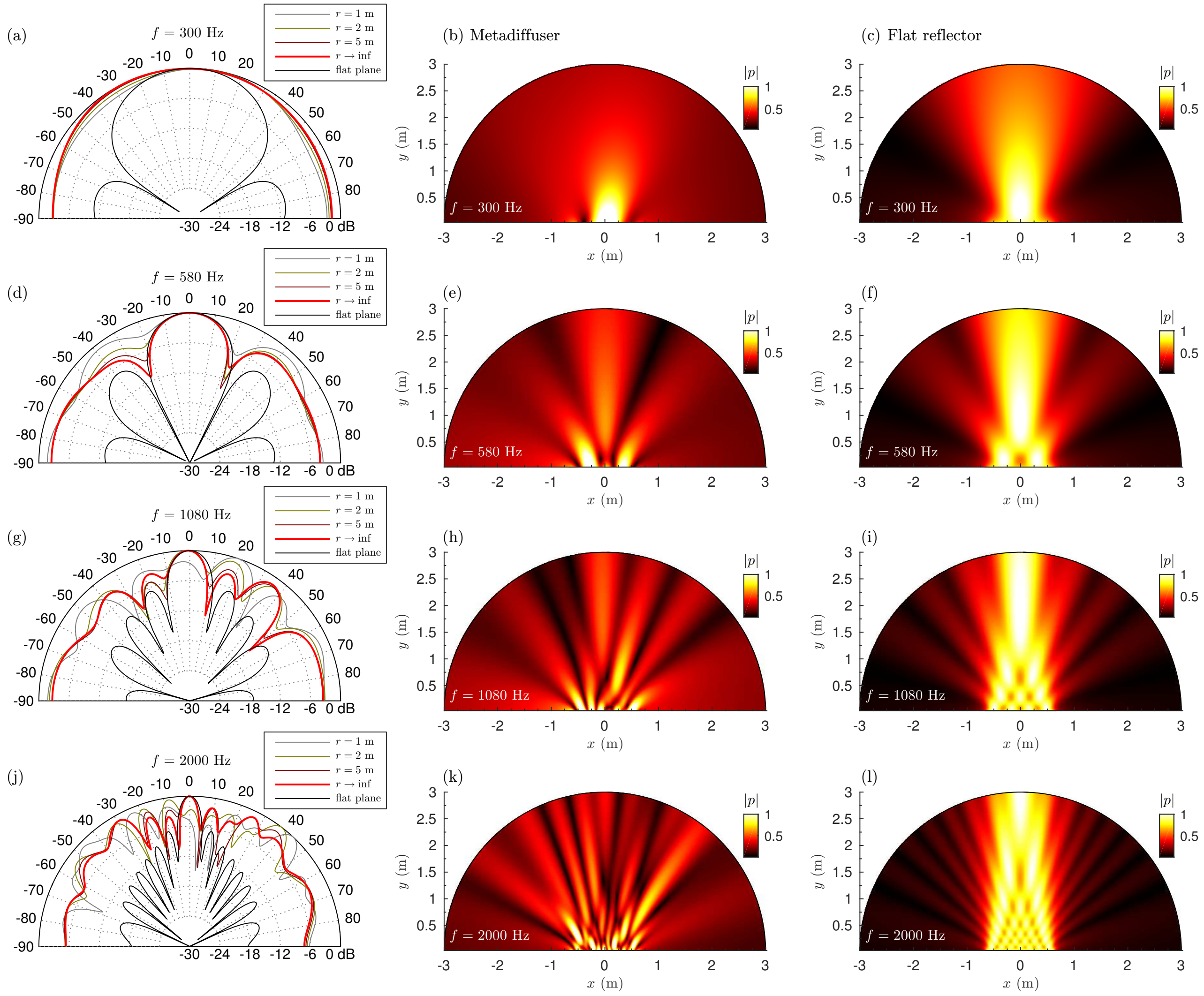

Figure 1. Polar responses and near field of the broadband metadiffuser. (a) Polar response of the broadband metadiffuser at 300 Hz, evaluated at 1 m from the structure (thin black), 2m (yellow), 5 m (dark red), far field response (thick red) and far field response of a flt reference plane with the same lateral dimension (thin black). (b) Map of the near field for the metadiffuser and (c) flat reference reflector. (d-f) corresponding polar and near field maps for 580 Hz, (g-i) 1080 Hz and (j-l) 2000 Hz. A single panel was considered.

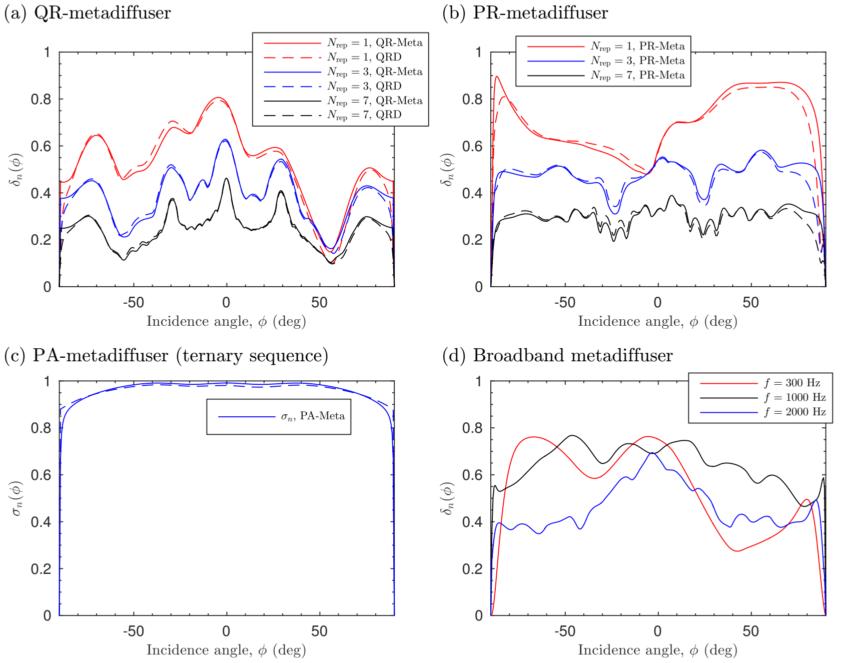

Figure 2. Performance of the diffusers as a function of the incidence angle, ϕ. (a) Normalized diffusion for the QR-metadiffuser (continuous lines) and the reference Schroeder's QRD (dashed lines) using $N_{\text{rep}} = 1,3,7$ repetitions of the panel (red, blue and black lines respectively). (b) Corresponding diffusion coefficient as a function of the incidence angle for the PR-metadiffuser (continuous lines) and the reference Schroeder's PRD (dashed lines). (c) Scattering coefficient, σ_n (blue), as a function of the incidence angle for the ternary PA-metadiffuser (continuous lines) and the ideal ternary sequence diffuser (dashed lines). (d) Diffusion coefficient as a function of the incidence angle for the broadband metadiffuser at 300 Hz (red), 1 kHz (black) and 2 kHz (blue).

dependence with the incidence angle, as it is shown in Fig. 2 (c). The scattering of an ideal ternary sequence diffuser agrees with the scattering of the PA-metadiffuser for all incidence angles. Only at grazing angles, when the magnitude of the reflection coefficient of these locally reacting surfaces increases, the scattering produced by the PA-metadiffuser is similar than a flat plane , i.e., $\sigma_n \to 0$.

Finally, the diffusion coefficient as a function of the incidence angle for the broadband metadiffuser is shown in Fig. 2 (d) for three frequencies. First, at 300 Hz the diffusion performance of the metadiffuser shows a strong dependence with the incidence angle, but, again, these variations are of the same order of a traditional design as both are locally reacting surfaces. At 1 kHz the response is more uniform with respect to the incidence angle. At 2 kHz, the product of the wavenumber, k, and the half size of the panel, $b = 0.66$ m, is high ($kb \approx 24$). Then, the reflection becomes more directive and this leads to a decreasing of the diffusion coefficient.

3 Parameter sensitivity

Changing the geometrical parameters of the metamaterial will change the resonance frequency of each slit and the amount of thermoviscous losses produced by the structure. A statistical parameter sensibility test was performed for the metadiffusers to study the impact of a variation of the geometrical parameters on the diffuser performance. A set of 10^4 structures were calculated where all their geometrical parameters were varied randomly up to 5% of its nominal values. A second set of structures were calculated using a 10% of variability of its geometrical parameters. Figures 3 (a,b) show the nominal, maximal and minimal bounds of the polar responses retrieved for the QR-Metadiffuser. Although differences between the diffuser performance can be observed, the main features of the QRD can be seen, e.g., the N grating lobes with almost same amplitude. The diffusion coefficient values, summarized in Table 1, present a maximal variation of 4% from their nominal values for a parameter variability of 5% and a maximal variation of 9% from its nominal value for a parameter variability of 10%.

Table 1. Summary of the parameter variability incidence on the diffusion coefficient d_n for the QR-metadiffuser.

parameter variability	mean d_n	std d_n	min bound d_n	max bound d_n
**5%** of the nominal values	0.54	0.0027	0.52	0.55
**10%** of the nominal values	0.54	0.0056	0.49	0.56

Same procedure was applied to the broadband diffuser. Figures 3 (c,d) show the frequency dependent diffusion coefficient for the broadband metadiffuser, where the parameters varied randomly, 5% and 10% of its nominal values, respectively. The maximal and minimal bounds (dashed lines) show roughly the same broadband behaviour. Table 2 summarizes the diffusion coefficient variability for three particular frequencies. The diffusion coefficient values, summarized in Table 1, present a

Table 2. Summary of the parameter variability incidence on the diffusion coefficient d_n for the Broadband metadiffuser.

parameter variability	mean d_n	std d_n	min bound d_n	max bound d_n
**5%** of the nominal values				
$f = 300$ Hz	0.79	0.020	0.75	0.81
$f = 1000$ Hz	0.65	0.003	0.63	0.64
$f = 2000$ Hz	0.58	0.007	0.54	0.56
**10%** of the nominal values				
$f = 300$ Hz	0.79	0.049	0.72	0.88
$f = 1000$ Hz	0.65	0.006	0.64	0.67
$f = 2000$ Hz	0.58	0.010	0.54	0.57

maximal variation of 5, 3, 7 % from their nominal values at for 300 Hz, 1 and 2 kHz, respectively, for a parameter variability of 5%. On the other hand, a maximal variation 9, 3, 7 % from their nominal values at for 300 Hz, 1 and 2 kHz, respectively, was observed for a parameter variability of 10%. The critical factors to these variations are the dimensions of the neck and the width of the slits, both key geometrical factors that introduce changes on the resonance frequency and the amount of thermoviscous losses of each slit. These results indicate that the parameter sensibility of metadiffusers is moderate up to errors in the construction of 10%.

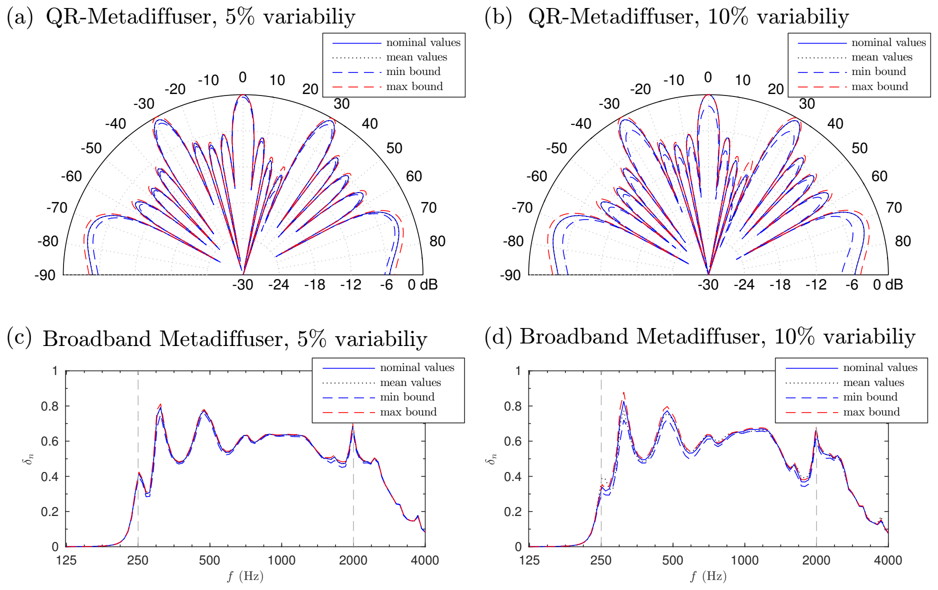

Figure 3. (a) (blue continuous) Diffusion coefficient of the QR-Metadiffuser for a parameter variability of 5%. Nominal values (blue), mean diffusion of the 10^4 calculation (dotted black), upper bound (dashed red), lower bound (dashed blue). (b) Corresponding diffusion for a 10% of the QR-metadiffuser. (c) Diffusion for a 10% variability of the geometrical parameters of the Broadband metadiffuser. (d) Corresponding diffusion for a 10% variability for the Broadband metadiffuser.

Acknowledgements

This article is based upon work from COST Action DENORMS - CA15125, supported by COST (European Cooperation in Science and Technology). The authors acknowledge financial support from the Metaudible Project No. ANR-13-BS09-0003, cofunded by ANR and FRAE.

Author contributions statement

N.J., V.RG. and JP.G. conducted the theoretical modelling and numerical experiment; N.J., V.RG. JP.G. and T.C. wrote the manuscript. All authors reviewed the manuscript.

Competing financial interests The authors declare no competing financial interests.
